# A taxonomy of epithelial human cancer and their metastases

**DOI:** 10.1186/1755-8794-2-69

**Published:** 2009-12-17

**Authors:** Olivier Gevaert, Anneleen Daemen, Bart De Moor, Louis Libbrecht

**Affiliations:** 1 Bioinformatics, Department of Electrical Engineering (ESAT/SCD), Katholieke Universiteit Leuven, Belgium; 2 Liver Facility and Laboratory of Hepatology, Department of Pathophysiology, Katholieke Universiteit Leuven, Belgium; 3 Department of Pathology, University Hospitals Gent, Belgium

## Abstract

**Background:**

Microarray technology has allowed to molecularly characterize many different cancer sites. This technology has the potential to individualize therapy and to discover new drug targets. However, due to technological differences and issues in standardized sample collection no study has evaluated the molecular profile of epithelial human cancer in a large number of samples and tissues. Additionally, it has not yet been extensively investigated whether metastases resemble their tissue of origin or tissue of destination.

**Methods:**

We studied the expression profiles of a series of 1566 primary and 178 metastases by unsupervised hierarchical clustering. The clustering profile was subsequently investigated and correlated with clinico-pathological data. Statistical enrichment of clinico-pathological annotations of groups of samples was investigated using Fisher exact test. Gene set enrichment analysis (GSEA) and DAVID functional enrichment analysis were used to investigate the molecular pathways. Kaplan-Meier survival analysis and log-rank tests were used to investigate prognostic significance of gene signatures.

**Results:**

Large clusters corresponding to breast, gastrointestinal, ovarian and kidney primary tissues emerged from the data. Chromophobe renal cell carcinoma clustered together with follicular differentiated thyroid carcinoma, which supports recent morphological descriptions of thyroid follicular carcinoma-like tumors in the kidney and suggests that they represent a subtype of chromophobe carcinoma. We also found an expression signature identifying primary tumors of squamous cell histology in multiple tissues. Next, a subset of ovarian tumors enriched with endometrioid histology clustered together with endometrium tumors, confirming that they share their etiopathogenesis, which strongly differs from serous ovarian tumors. In addition, the clustering of colon and breast tumors correlated with clinico-pathological characteristics. Moreover, a signature was developed based on our unsupervised clustering of breast tumors and this was predictive for disease-specific survival in three independent studies. Next, the metastases from ovarian, breast, lung and vulva cluster with their tissue of origin while metastases from colon showed a bimodal distribution. A significant part clusters with tissue of origin while the remaining tumors cluster with the tissue of destination.

**Conclusion:**

Our molecular taxonomy of epithelial human cancer indicates surprising correlations over tissues. This may have a significant impact on the classification of many cancer sites and may guide pathologists, both in research and daily practice. Moreover, these results based on unsupervised analysis yielded a signature predictive of clinical outcome in breast cancer. Additionally, we hypothesize that metastases from gastrointestinal origin either remember their tissue of origin or adapt to the tissue of destination. More specifically, colon metastases in the liver show strong evidence for such a bimodal tissue specific profile.

## Background

Microarray technology has allowed to molecularly characterize many different types of cancer [1]. One of the first landmark studies using microarray technology to analyze primary tumor samples was done by Golub *et al*. [2]. This study on human acute leukemia demonstrated that it was possible to use microarray data to distinguish acute myeloid leukemia from acute lymphoblastic leukemia without any previous knowledge. The authors showed for the first time the potential of microarray technology by illustrating its use in discovering new classes and by using microarray data to assign tumors to known classes. Class prediction gives the clinician an unbiased method to predict the outcome of cancer patients in comparison to traditional methods based on histopathology or empirical clinical data, which do not always reflect patient outcome. More recently, for some cancer sites these initial discoveries have been validated in independent data sets [3-5]. This and other initial applications of microarray technology primarily focused on discovering molecular subtypes within each cancer site using only samples from the primary tumor site [6-9].

Other groups focused on tissue specific differences between cancer sites by building supervised models that classify samples according to their tissue of origin [10,11] or by comparing cancer from multiple tissues with normal tissue [12]. In a landmark study by Ramaswamy *et al*. the expression profile of primary and metastatic adenocarcinoma of diverse origins was compared and they found that a signature distinguishing primary and metastatic tumors was also active in many primary tumors [13]. This signature proved to be significantly correlated with metastasis and poor clinical outcome in independent data sets. In a similar study Glinksy *et al*. developed an 11-gene signature that was predictive of a short interval to disease recurrence, distant metastasis, and death after therapy in cancer patients diagnosed with many types of cancer [14]. Also Rhodes *et al*. have performed a meta-analysis by comparing the expression profiles of many types of cancers with normal tissue from many published studies. They concluded that a common transcriptional program exists characterizing neoplastic transformation [12].

These studies indicated that the primary site can potentially be predicted for cancer of unknown origin. This is an important issue for clinicians since in 3-5% of cancer cases the primary tissue is unknown. This is often called cancer of unknown primary (CUP) [15] and many efforts have been done to find ways to predict the primary site based on microarray data. Reported performances are in the range of 70-90% accuracy [16-20]. Overall these studies have shown that many metastatic tumors "remember" their tissue of origin.

These studies demonstrated that microarray technology can molecularly characterize cancer and its enormous heterogeneity when discovered in multiple tissues. However, due to technological differences and issues in standardized sample collection, no study in a large number of samples and tissues has been done to molecularly profile both primary and metastatic epithelial cancer in an unbiased way. For primary tumors, previous studies focused on a single cancer site [2-9] or compared a limited number of tumors from a limited number of cancer sites [10,11]. Additionally, an extensive investigation whether metastases resemble their tissue of origin or tissue of destination has not been performed. The previously mentioned CUP studies have shown that tissue of origin can be predicted with reasonable accuracy; however, none of these studies have reported misclassifications of their signatures in detail and whether they are tissue specific.

In this contribution, we studied the expression profiles of a series of 1566 primary tumors and 178 metastases of different tissues gathered in the framework of the expression project for oncology (expO) project by the international genomics consortium. We used unsupervised analysis to identify, in an unbiased way, the relationships between primary tumors and their metastases. The clustering profile was subsequently investigated and extensively correlated with clinico-pathological data. Our results reveal relationships between cancers in different tissues, show the existence of new molecular subgroups across tissues and we found a signature predictive of clinical outcome. Moreover, our results on the behavior of metastases of epithelial human cancer can have important consequences for the treatment of CUP and its associated research.

## Methods

### Data

We used data from the expression project for oncology (expO) gathered by the International Genomics Consortium to investigate the molecular differences between primary epithelial tumors and their metastases. The expO project started in 2004 and new data is still being added to the repository. We used data from the batches 1 to 16 (December 2008) which amounts to 2173 microarrays in 142 different cancer sites extracted from GEO (GSE2109) [21]. We selected 1566 primary epithelial tumors from 18 cancer sites (see Table 1 and Table 2) and 178 metastases of similar primary cancer sites, metastasizing to over 40 different tissues or anatomical sites. Non-epithelial cancers were not included since their numbers were rather low and their etio-pathogenesis is essentially different from that of epithelial cancers. Tissues were not excluded based on a small number of samples.

**Table 1:** number of primary tumor samples in each cluster

Primary tumors									
**Primary tumors**	**Breast cluster**	**Colon cluster**	**Lung cluster**	**Ovary cluster**	**Kidney cluster**	**Prostate cluster**	**Thyroid Kidney cluster**	**Mix cluster**	**Total**

**bladder**	6	1	10	11	0	0	0	0	**28**
**breast**	331	3	4	3	4	0	2	6	**353**
**cervix**	3	8	18	0	1	0	0	1	**31**
**colon**	14	254	6	2	1	0	1	1	**279**
**endometrium**	1	2	1	52	0	0	1	6	**63**
**fallopian tube**	0	0	0	0	0	0	0	0	**0**
**kidney**	7	0	0	0	248	0	20	3	**278**
**liver**	0	1	2	0	9	1	0	1	**14**
**lung**	4	0	107	5	0	0	4	1	**121**
**ovary**	2	9	7	147	0	0	1	9	**175**
**pancreas**	0	0	0	0	0	0	0	0	**0**
**peritoneum**	0	0	0	0	0	0	0	0	**0**
**prostate**	1	0	2	0	0	80	0	0	**83**
**rectosigmoid**	1	30	0	0	0	0	0	0	**31**
**rectum**	5	30	1	0	0	0	0	0	**36**
**renal pelvis**	2	0	2	3	0	0	1	0	**8**
**small intestine**	1	3	1	0	0	0	1	1	**7**
**stomach**	0	8	1	0	0	0	0	2	**11**
**testis**	0	0	0	0	0	0	0	0	**0**
**thyroid**	1	2	5	0	1	0	22	2	**33**
**uterus**	1	0	0	2	0	0	0	2	**5**
**vulva**	1	0	9	0	0	0	0	0	**10**

**Total**	**381**	**351**	**176**	**225**	**264**	**81**	**53**	**35**	**1566**

**Table 2:** number of metastatic tumor samples in each cluster, with the metastases represented according to their primary tissue

Metastatic tumors									
**Primary tissues**	**Breast cluster**	**Colon cluster**	**Lung cluster**	**Ovary cluster**	**Kidney cluster**	**Prostate cluster**	**Thyroid Kidney cluster**	**Mix cluster**	**Total**

**bladder**	0	0	1	0	0	0	0	0	**1**
**breast**	5	1	0	0	0	0	0	0	**6**
**cervix**	0	0	2	1	0	0	0	0	**3**
**colon**	1	18	2	5	10	0	1	0	**37**
**endometrium**	4	2	1	9	0	0	0	4	**20**
**fallopian tube**	0	0	0	5	0	0	0	0	**5**
**kidney**	0	0	1	0	1	0	0	0	**2**
**liver**	0	0	0	0	0	0	0	0	**0**
**lung**	0	1	4	1	0	0	0	0	**6**
**ovary**	6	0	1	63	1	1	0	5	**77**
**pancreas**	1	1	1	0	0	0	0	0	**3**
**peritoneum**	0	0	0	2	0	0	0	0	**2**
**prostate**	0	0	0	0	0	0	0	0	**0**
**rectosigmoid**	0	1	0	1	1	0	0	0	**3**
**rectum**	0	2	0	0	1	0	0	0	**3**
**renal pelvis**	0	0	0	0	0	0	0	0	**0**
**small intestine**	0	0	1	0	0	0	0	1	**2**
**stomach**	0	3	0	0	0	0	0	0	**3**
**testis**	1	0	0	0	0	0	0	0	**1**
**thyroid**	0	0	0	0	0	0	0	0	**0**
**uterus**	0	0	1	0	0	0	0	2	**3**
**vulva**	0	0	1	0	0	0	0	0	**1**

**Total**	**18**	**29**	**16**	**87**	**14**	**1**	**1**	**12**	**178**

### Preprocessing

The tumors were profiled using the Affymetrix GeneChip Human Genome U133 Plus 2.0 Array. Due to the size of the complete data set, preprocessing was done using the simpleaffy implementation of the MAS5 preprocessing algorithm [22]. We used a custom cdf file developed by Manhong Dai and colleagues (version 10 May 2007, Hs133P_Hs_ENTREZG) such that probe sets are up-to-date with the genome sequence and correspond to Entrez gene identification [23]. Next, to check whether similar genes were called expressed in both primary and metastases tumors, we selected genes that were present in 80% of the samples in each set separately before merging both data sets.

### Unsupervised modeling

To facilitate interpretability of the clustering, the 250 genes with the highest variance were selected from the data set. Unsupervised modeling was subsequently performed using hierarchical clustering with the Euclidean distance for calculating the similarity between genes and the cosine distance for the similarity between samples using average linkage. Matlab version R2009b and the bioinformatics toolbox from Matlab version 3.4 were used for hierarchical clustering.

### Statistical analysis

Statistical enrichment of clinico-pathological annotations of groups of samples was investigated using Fisher exact test. All reported p-values are based on Fisher exact test unless otherwise reported. Gene set enrichment analysis (GSEA) and DAVID functional enrichment analysis were used to investigate the molecular pathways, enriched in differentially expressed gene lists between groups of tumors [24-26]. Kaplan-Meier survival analysis and log-rank tests were used to investigate prognostic significance of gene signatures using SAS version 9.1. Differential expression analysis was done using the wilcoxon rank sum test to rank genes.

## Results

### Preprocessing

After preprocessing each sample separately using the simpleaffy implementation of MAS5, only probes with a present call in 80% of the samples were retained [22]. This was done separately for the set of primary and metastatic tumors. Additionally, updated annotation files based on reorganizing probes to Entrez gene specific probe sets excluding inaccurate or wrongly annotated probes was used to annotate the Affymetrix probes [23]. This resulted in 7732 and 7689 genes out of 17527 that were called present in the primary and metastatic data set respectively. 7504 genes overlapped between these two sets, indicating that similar genes have present calls in primary and metastatic tumors.

### Primary tumors

**Figure 1:**
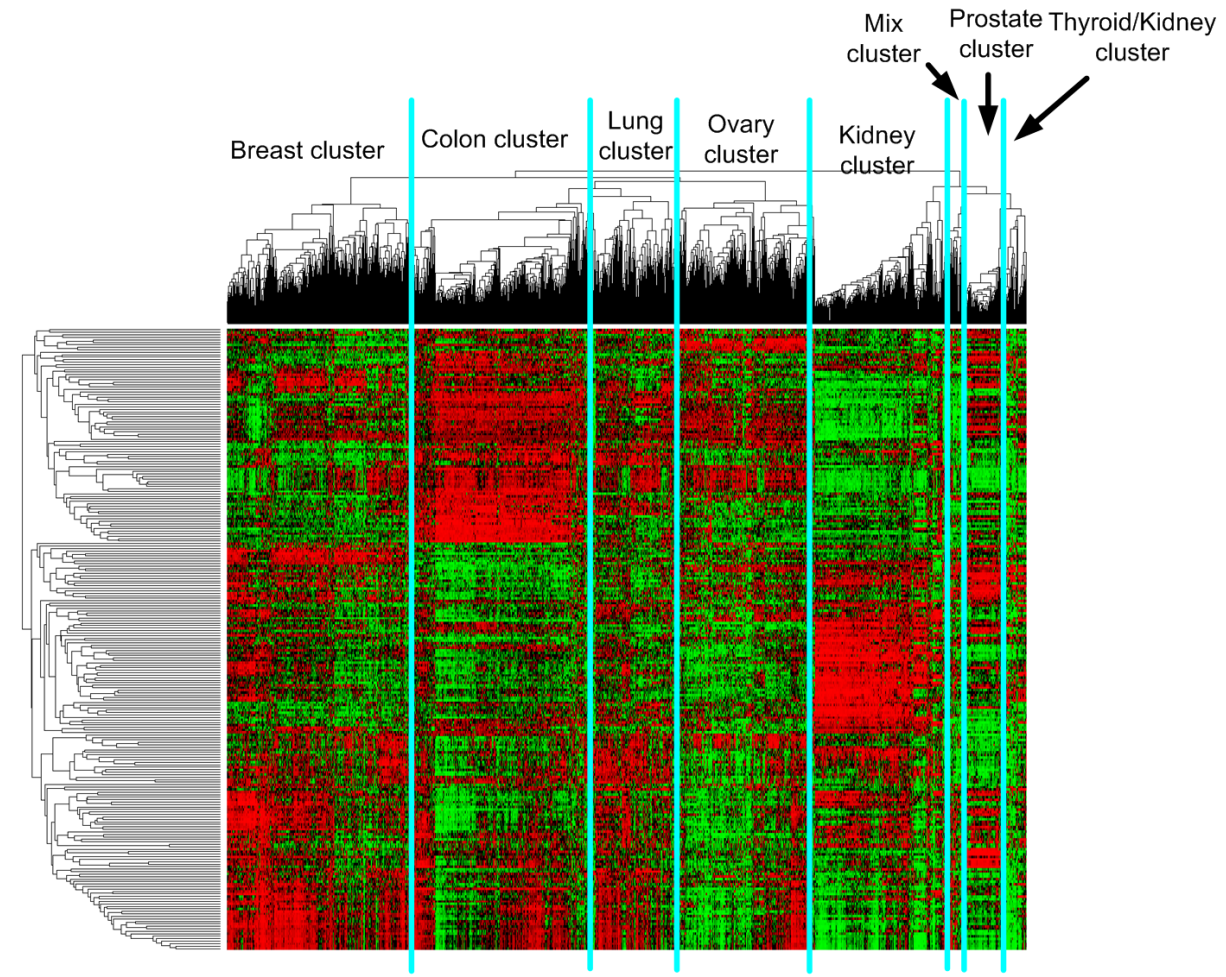
**Hierarchical clustering of 1566 primary epithelial human cancer tumors and 178 metastatic tumors of epithelial origin**.

**Figure 2:**
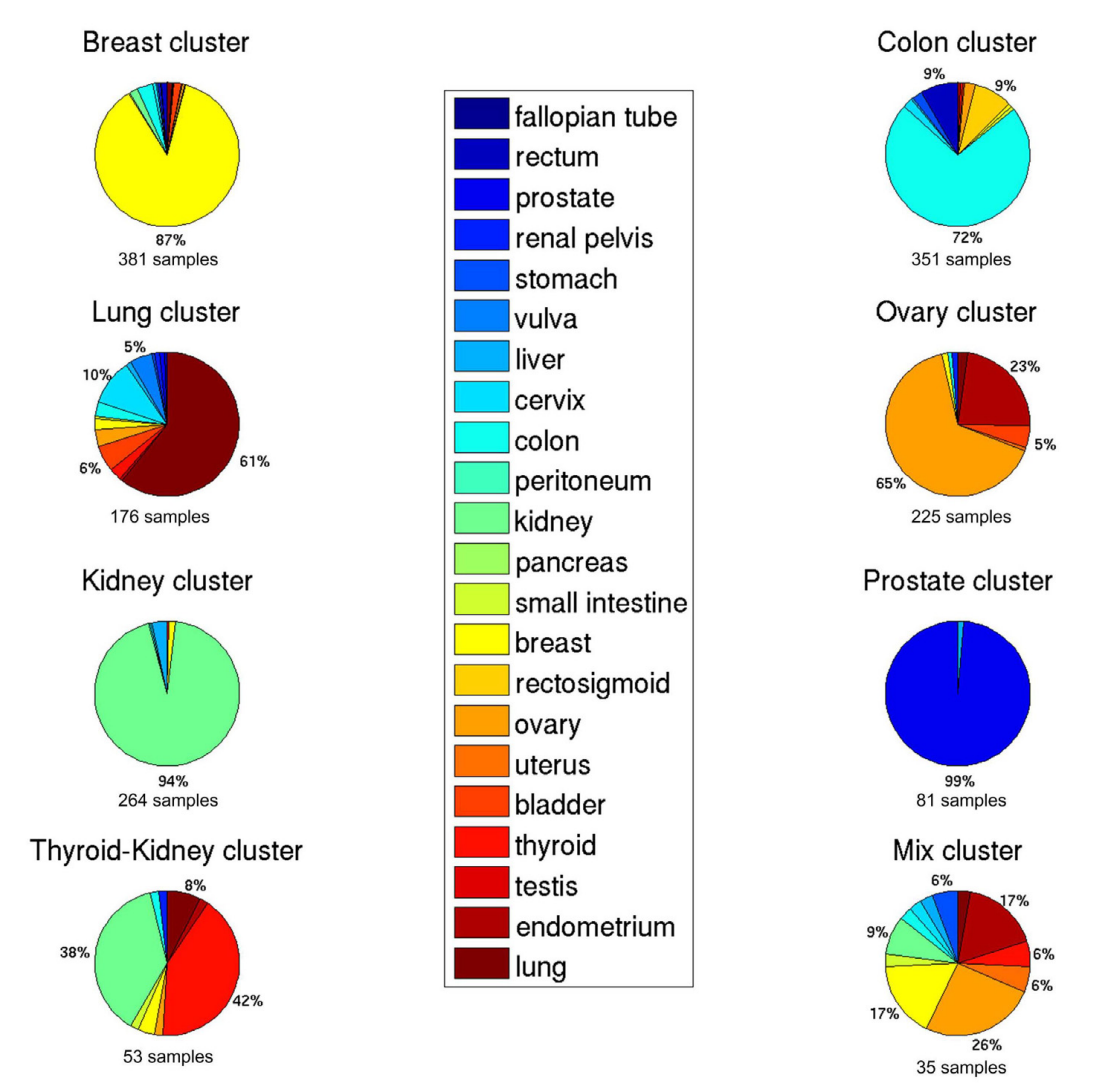
**Cluster composition for the primary tumor samples**. The total number of primary samples in each cluster is indicated.

**Figure 3:**
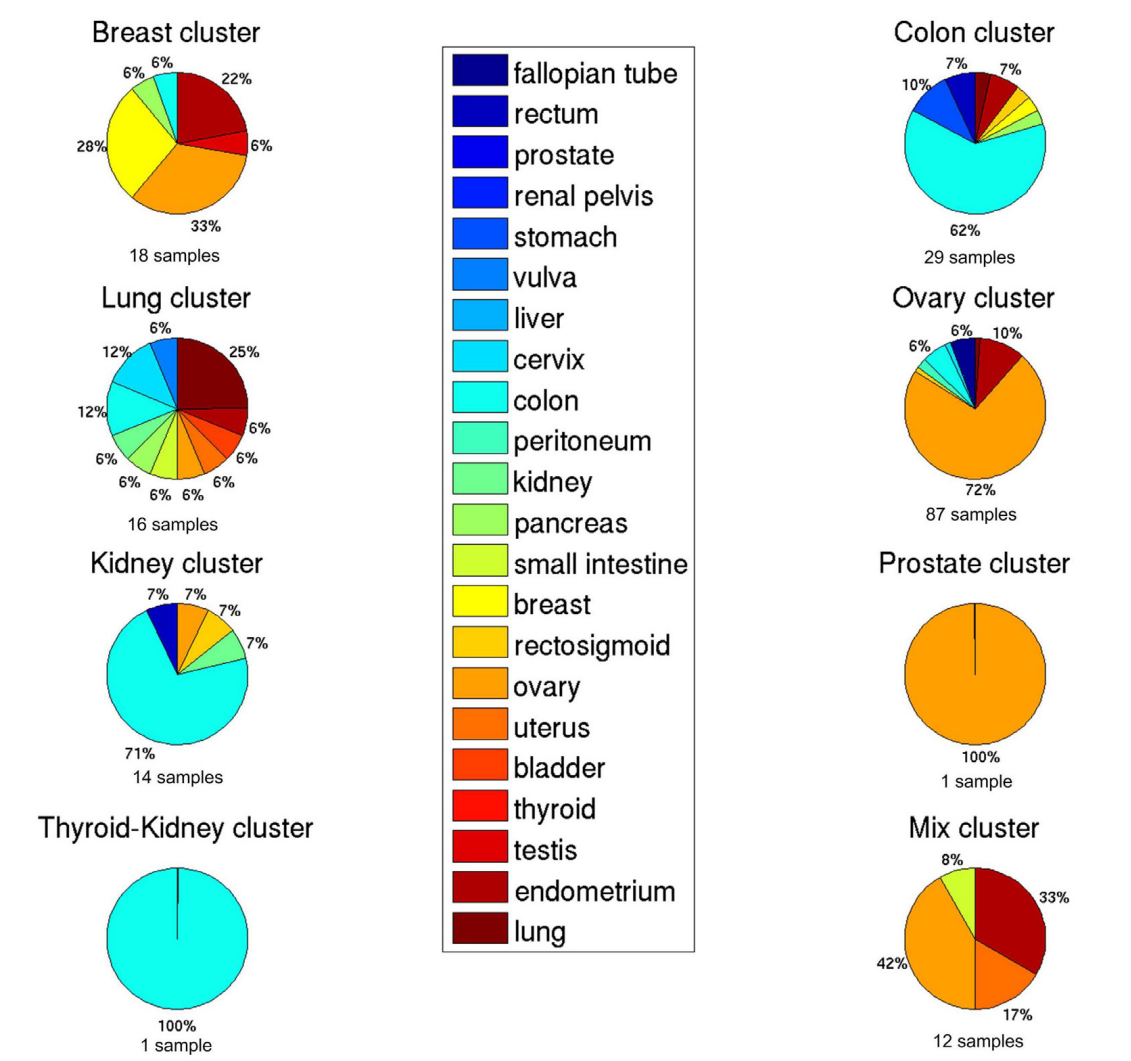
**Cluster composition for the metastatic tumor samples according to their primary site**. The total number of metastases samples in each cluster is indicated.

Figure 1 shows the clustering of all primary and metastatic tumors using the top 250 genes with the largest variance over all samples. Five large groups can be distinguished enriched for primary breast, colon, lung, ovary and kidney tissues. In addition three smaller clusters can be distinguished corresponding to a prostate cluster, a thyroid-kidney cluster and a cluster with mixed tissues. We have named each cluster according to its enriched primary tissue. Figure 2 and Figure 3 show the composition of each molecular cluster, separately for primary and metastatic tissues (see also Table 1 and 2 for the complete composition of the clusters and Additional File 1 for a list of samples in each cluster).

Table 1 and Figure 1 show that the prostate cluster is the most homogeneous cluster compared to all other clusters. 98% (80/82) of the samples in this cluster are primary prostate tumors and only 3.6% (3/83) of primary prostate samples do not cluster here, indicating that prostate tissue is very different from all other tissues. This is further supported by gene set enrichment analysis (GSEA) analysis since a set of genes upregulated by androgen in neoplastic prostate epithelium [27] is the most significantly expressed gene set in this cluster compared to all other clusters (See Additional File 2).

**Figure 4:**
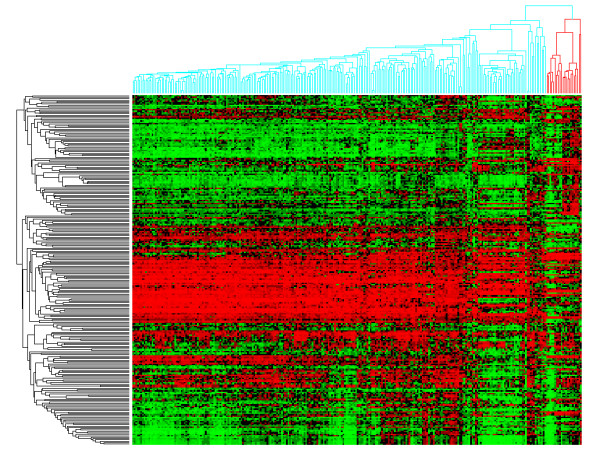
**Kidney subclustering**.

The kidney cluster is the second most homogeneous cluster consisting of 89% (248/278) primary kidney tumors. Additionally, only 30 primary kidney tumors do not cluster here. This cluster primarily expresses pathways related to hypoxia and cytokine receptor interaction when compared to the other clusters (See Additional File 2). The kidney cluster also contains a primary liver subcluster; 64% (9/14) of primary liver tumors cluster here (See Figure 4).

**Figure 5:**
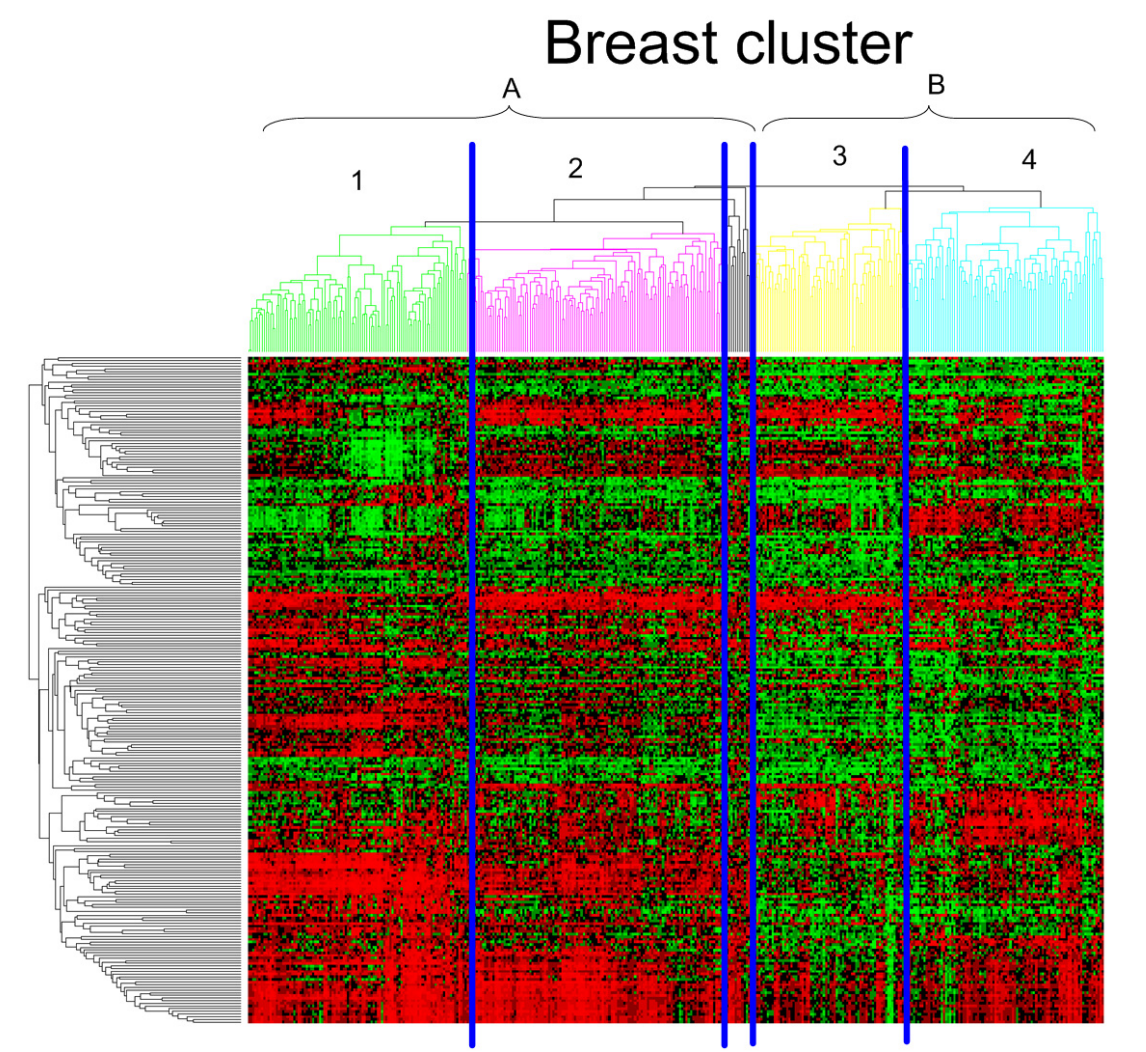
**Breast subclustering**.

Next, the breast cluster contains a significant portion of the primary breast tumors (84% or 331/353). However, this cluster is less homogeneous with 83% (331/399) of the tumors in this molecular cluster being primary breast samples. A more detailed analysis shows that these breast tumors are subdivided according to histology and grade. The left branch is a mixed lobular-ductal cluster containing 87% (33/38, P-value < 0.000009) of the lobular carcinoma while the right branch is a mainly ductal cluster (89% or 136/152) (Clusters A and B in Figure 5, respectively). It should be mentioned that all lobular carcinomas in the expO data set are of the classical, non-pleomorphic type. Overall, the ductal breast carcinoma are approximately equally divided over both subclusters (48% in lobular-ductal vs. 52% in the pure ductal subcluster). However, the pure ductal cluster is enriched for grade 3 while the mixed lobular-ductal cluster contains mostly grade 1 and 2 primary breast tumors (see Additional File 3).

On the next level in the hierarchical tree, both cluster A and B separate in two distinct clusters (clusters 1-2, and clusters 3-4, respectively, see Figure 5). Cluster 1 contains the highest concentration of lobular tumors. Clusters 3 and 4 are separated according to receptor status. Cluster 4 is enriched with triple negative tumors (ER, PR and ERBB2 negative) (71% or 32/45, Pvalue < 4.74e-8), while Cluster 3 has similar receptor positivity as the remaining breast tumors in Cluster A.

**Figure 6:**
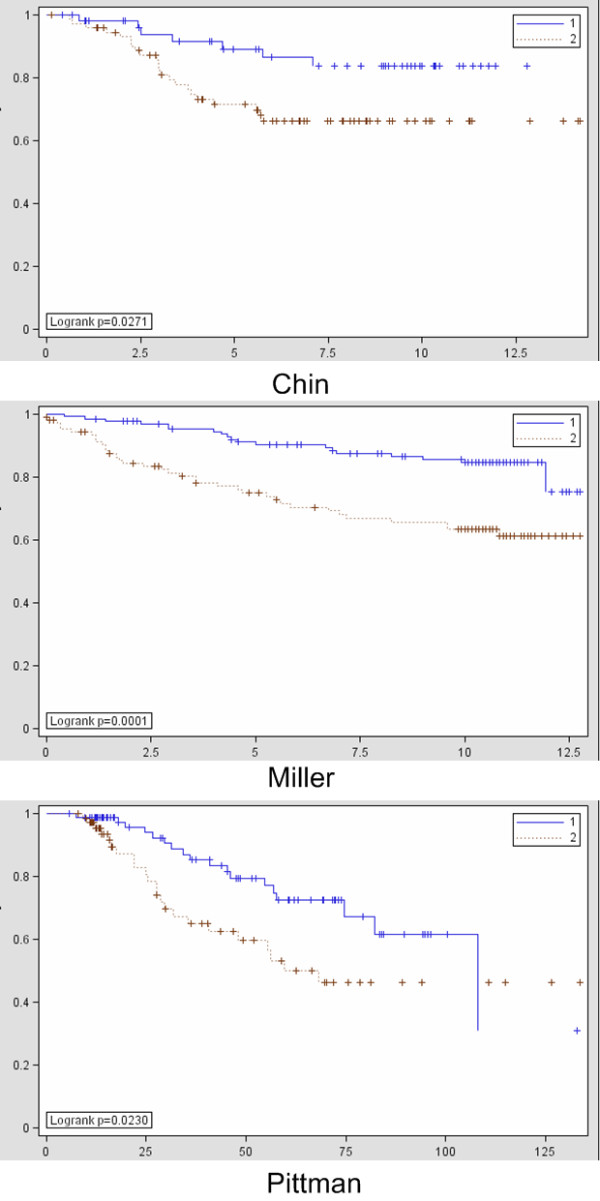
**Investigation of the prognostic relationship of the breast clustering signature in three external data sets with 1 = good prognosis group and 2 = poor prognosis group**.

The association of the clusters with histology, tumor grade and receptor status indicated a possible relationship with breast cancer prognosis. Therefore, we investigated whether differential expression between subgroups contains prognostic information. Starting from all genes, we selected the 250 most differentially expressed genes between Cluster 1 and Cluster 4 and used this set of genes as a prognostic signature (see Figure 5 and Additional File 4). Cluster 1 contains the highest concentration of lobular tumors with lowest grade, while Cluster 4 is purely ductal, high grade and contains most triple negative tumors. We used three external data sets to investigate the ability of this signature to distinguish between prognostic groups by clustering patients with the signature genes and using the first split in the hierarchical tree as prognostic groups (See Table 3). In all three data sets comprising 539 patients in total, the signature was significantly predictive for disease specific survival with p-values of 0.0271, 0.0001 and 0.0230 for the Chin, Miller and Pittman data sets, respectively (log-rank test, see Figure 6).

**Table 3:** data sets used for investigating the prognostic relationship of the breast cluster signature

Data set name	Number of patients	Microarray type	Outcome
Chin [72]	130	Affymetrix GeneChip Human Genome U133A Array Set	Disease specific survival (Event = death from breast cancer)

Miller [73]	251	Affymetrix GeneChip Human Genome U133 Array Set	Disease specific survival (Event = death from breast cancer)

Pittman [74]	158	Affymetrix GeneChip Human Genome U95av2 Array Set	Overall survival (Even t = death from breast cancer)

**Figure 7:**
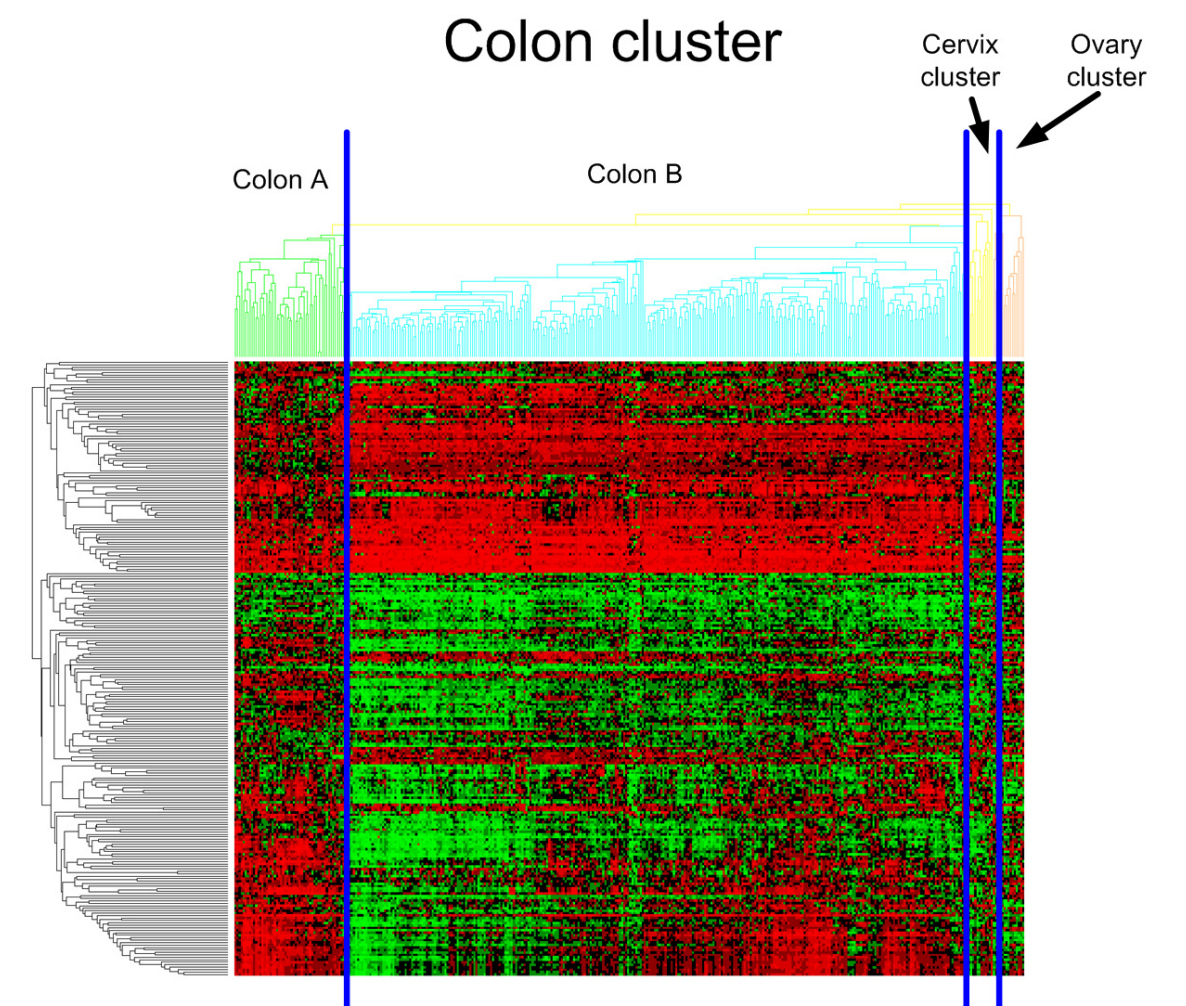
**Colon subclustering**.

The following cluster, the colon cluster, contains 91% (254/279) of colon samples which defines 67% (254/380) of this cluster. In addition, this cluster is enriched for all other primary tumors of gastrointestinal origin since it contains 97% (30/31) of all primary rectosigmoid tumors, 83% (30/36) of the primary rectum tumors, 72% (8/11) of the stomach tumors and 43% (3/7) of the primary small intestine tumors. Taken together 325/364 tumors (89%, Pvalue < 5.58e-255) of gastrointestinal origin are in this molecular cluster. When investigating the subclustering within this colon cluster in more detail, a small and a large colon subcluster, which we will refer to as Colon A and Colon B (See Figure 7), appear from the data. When focusing solely on the primary colon samples in these cluster, Colon A is enriched for high grade tumors (Grade >= 3, Pvalue 9.71e-05) and positive lymph nodes (N >0, Pvalue 0.012) when compared to Colon B. There was no significant relationship for tumor stage and histology between both groups.

Next, the lung cluster contains 88% (107/121) of primary lung tumors but is the least homogeneous cluster containing 44% (85/192) other primary tumors. This includes 90% (9/10) of primary vulva tumors and a significant portion of primary cervix tumors (58% or 18/31). This is most likely due to the enrichment of the squamous cell carcinoma histology in this cluster. The cervix tumors in this cluster are enriched for squamous cell carcinoma (16/18 are squamous cell carcinoma), compared to the cervix tumors in the colon cluster containing no squamous cell carcinoma (0/8). Similarly, all vulva samples in this cluster are of the squamous type (9/9). Taken together, the lung cluster is highly enriched for the squamous cell carcinoma histology since 38% (66/176) of tumor samples are of this type and of different tissues (i.e. bladder, cervix, lung and vulva) but more importantly 83% (66/80, Pvalue < 3.66e-54) of all squamous tumors cluster here.

The ovary cluster consists of 84% (147/175) of the primary ovarian tumor samples which make up 47% (147/312) of this cluster. In addition, 83% (52/63) of the endometrium tumors cluster here. More specifically, the ovarian cluster is divided into two subclusters: an endometrioid-enriched cluster and a serous-enriched cluster. The former contains all endometrium tumors and 88% (22/25, P-value < 0.00015) of pure endometrioid ovarian tumors.

The 28 bladder tumors are spread over three different clusters: breast, lung and ovary. 39% (11/28) cluster in the endometrioid subcluster of the ovary cluster. Most bladder tumors in the expO data set are transitional cell carcinoma (TCC). TCC of the ovary also exist [28,29] and occurred twice in our data set, both clustered in the same endometrioid subcluster possibly explaining why a significant part of bladder tumors clusters together with the endometrioid ovaries.

Finally, the thyroid/kidney cluster contains a significant amount of thyroid (41% or 22/54) and kidney samples (37% or 20/54). The subgroup of kidney tumors that clusters with thyroid tumors rather than in the kidney cluster is enriched for the chromophobe histology (P-value < 6.3e-8). In addition, when ignoring the kidney tumors from the granular cell carcinoma histology since this is a nonspecific, outdated descriptor [30], the enrichment is even more significant with 9/13 of the remaining kidney tumors being chromophobe (P-value < 2.5e-9). In addition, the thyroid tumors in this cluster are more frequently follicularly differentiated (9/22) compared to the thyroid tumors in other clusters (3/11); however, not significantly due to the low number of thyroid tumors of follicular differentiation. In addition, all non-papillary follicular thyroid tumors cluster here. GSEA analysis on the thyroid-looking kidney samples vs. the kidney samples in the kidney cluster reveals that gene sets related to oxidative phosphorylation and mitochondrion are upregulated in this subset of thyroid-looking kidney tumors (see Additional File 5).

### Metastases

To investigate whether metastases cluster with tissue of origin or destination, we assigned each tissue to a cluster where it was most significantly enriched with its corresponding primary tumors. Then we investigated if a metastatic tumor clusters with its tissue of origin or tissue of destination. When a tissue was enriched in multiple clusters we did not investigate metastases of this tissue, which was the case for the cervix and bladder tissues.

Metastases originating from breast (P-value < 0.003), lung (P-value < 0.002), cervix (P-value < 0.034), endometrium (P-value < 0.004), stomach (P-value < 0.010) and ovarian (P-value < 2.8e-36) are significantly enriched in their tissue of origin cluster. The latter, the metastases of ovarian origin, are more specifically enriched in the serous ovarian sub cluster (P-value < 1.72e-13). In addition, one vulva-to-liver metastasis clusters with the primary vulva tumors and all fallopian tube and peritoneum metastatic tumors; although both tissues are not represented with primary tumors, cluster in the most likely related ovarian molecular cluster. Together, this indicates that metastases from these tissues "remember" their tissue of origin and reflect the original tissue in their molecular profile.

Exceptions to this rule are metastatic tumors arising from gastrointestinal origin such as colon, rectum and rectosigmoid, where a bimodal distribution is seen. A significant part of these tumors cluster with the tissue of origin while another part clusters with the tissue of destination. For example, for the metastases from colon, 49% (18/37) cluster in the colon molecular cluster while metastasizing to different sites (i.e liver, omentum, ovary, bladder and lung). However, 14% (5/37) cluster in the ovary cluster enriched for colon to ovary metastases (P-value < 0.02) and 27% (10/37) cluster in the kidney/liver cluster enriched for colon to liver metastases (P-value < 0.01). A similar result for a much smaller group and thus not significant, is seen for three colon-to-lung metastases of which two tumors cluster in the colon cluster and one in the lung cluster.

Moreover, similar results are seen for smaller groups of tumors in the rectosigmoid and rectum site. Two rectum-to-liver metastases cluster in the colon cluster while the remaining rectum-to-liver metastases clusters in the kidney/liver cluster. One of the rectosigmoid metastases clusters in the colon cluster while the other two, a rectosigmoid to ovary and a rectosigmoid to liver metastasis cluster in the ovary and kidney/liver cluster, respectively.

Because the colon-to-liver metastases are the largest group of tumors within this class, we focused on this subset for a more detailed analysis. We used GSEA to investigate the molecular differences between the colon-to-liver metastases that cluster in the colon cluster (9/20) vs. the colon-to-liver metastases that cluster in the kidney/liver cluster (9/20). Additional File 6 shows the significantly upregulated pathways in the colon and liver subgroups. Interestingly, a set of liver specific genes is upregulated in the liver subgroup indicating that these colon-to-liver metastases indeed adapt to the liver tissue. Additionally, a set of genes upregulated in hepatocellular carcinoma (HCC) of good survival is also upregulated in the liver subgroup while the gene set corresponding to poor survival in HCC is upregulated in the colon subgroup. In addition, gene sets related to well known metabolic processes in the liver are significantly upregulated in the colon-to-liver metastases clustering with the primary liver tumors.

## Discussion

Our results show interesting correlations between tissues and clinicopathological variables such as stage, grade or histology. Now, we will discuss the most compelling results for each cluster.

### Prostate cluster

This cluster is clearly the most homogeneous one. Since only 16% of tumors in this cluster were low-grade (i.e. Gleason score <7), this homogeneity can not be explained by the assumption that most of these tumors are well differentiated and form a very distinct cluster based on the high expression of prostate-specific genes as such. In contrast with most other epithelial tumors, prostate cancer is characterized by little or no desmoplastic reactive stroma [31]. Thus, the homogeneity can be explained by the fact that in a sample of prostate cancer the expression of tissue-specific genes by epithelial tumor cells is less 'contaminated' by the stroma compared to samples of epithelial tumors of other organs. As further discussed in the breast cluster, this underscores that the role of tumor stroma has to be taken into account when evaluating molecular data from non-microdissected samples.

### Kidney cluster

The kidney cluster mainly expressed hypoxia related genes. This corresponds to a large body of research that has shown that loss of the VHL gene activates HIF resulting in uncontrolled angiogenesis in the kidney [32]. Moreover, it has been shown that loss of VHL is connected to CXCR4 up-regulation implicating the cytokine receptor pathway [33]. Our results confirm this since both hypoxia related pathways and the cytokine receptor pathway are over-expressed in the kidney cluster. In addition the kidney cluster is enriched for clear-cell renal carcinoma (P-value < 0.002) which has been shown to be caused by loss of VHL.

The similarity between liver and kidney tumors is striking (See Figure 4) and seems to be caused by similar genes defining the liver and kidney tissues. This can be explained due to a significant overlap between liver and kidney specific genes based on tissue expression profiles from the TIGER database compared to other tissue comparisons (see Additional File 7) [34]. More specifically, 38 genes overlap between liver and kidney specific genes. In addition, all liver tumors in this cluster are hepatocellular carcinoma (HCC) of grade 2 and low stage (i.e., <= 2), possibly indicating that their tissue specific profile has not been significantly scrambled by oncogenic processes. On the other hand, the remaining HCC are of high stage and grade (i.e., >= 3) and appear to cluster randomly, possibly indicating a loss of primary tissue profile associated with grade (see Table 1).

### Breast cluster

The breast cluster is subdivided according to histology in a mixed lobular-ductal cluster with mainly low and intermediate grade tumors and a ductal-enriched cluster with mainly high grade tumors. This confirms the hypothesis that non-pleomorphic lobular breast carcinoma can be considered as a low-grade subtype of ductal breast tumors; only the status of CDH1 expression is strongly different between the two types, which causes strong morphological differences [35-37]. When comparing the lobular and ductal tumors in the lobular-ductal cluster (Cluster A), CDH1 is the most significantly differential gene and upregulated in the lobular tumors (see Additional File 8).

Moreover, the clear association with tumor grade potentially indicates that the molecular differences between the two subtypes have prognostic implications. We therefore investigated the prognostic significance of a gene signature differentially expressed between the hypothesized good and poor prognosis groups. The positive external validation of the signature confirms that classical, non-pleomorphic lobular are related to good prognosis while ductal tumors appear in both prognostic groups but can be separated according to grade. In addition, the triple negative receptor status in Cluster 4 is confirmed as having a negative prognostic impact [38,39]. Our signature is robust since it could be validated in breast cancer data sets that were heterogeneous regarding grade, stage and ER-status.

The association of grade with clinical outcome has already been confirmed by others [40,41].

More specifically, the group of Sotiriou has shown that the performance of prognostic signatures is due to the presence of proliferation-related genes [42]. Our signature shows an overlap of 23 genes with the Genomic Grade Index (GGI) of Sotiriou [40]. As it has previously been shown that most signatures studying the same disease and outcome share few genes but more pathways [43], a pathway analysis was performed. These results show significant overlap with the GGI, highlighting proliferation pathways such as mitosis, cell cycle and cell division which are highly expressed in Cluster 4 (see Additional File 9). However, when focusing on the genes over-expressed in the lobular enriched cluster (Cluster 1), other pathways seem to characterize the good prognosis samples. These include genes related to or located in the extracellular matrix, secreted genes and genes containing the EGF domain (see Additional File 9). The latter are present in a large number of membrane-bound and extracellular proteins.

**Figure 8:**
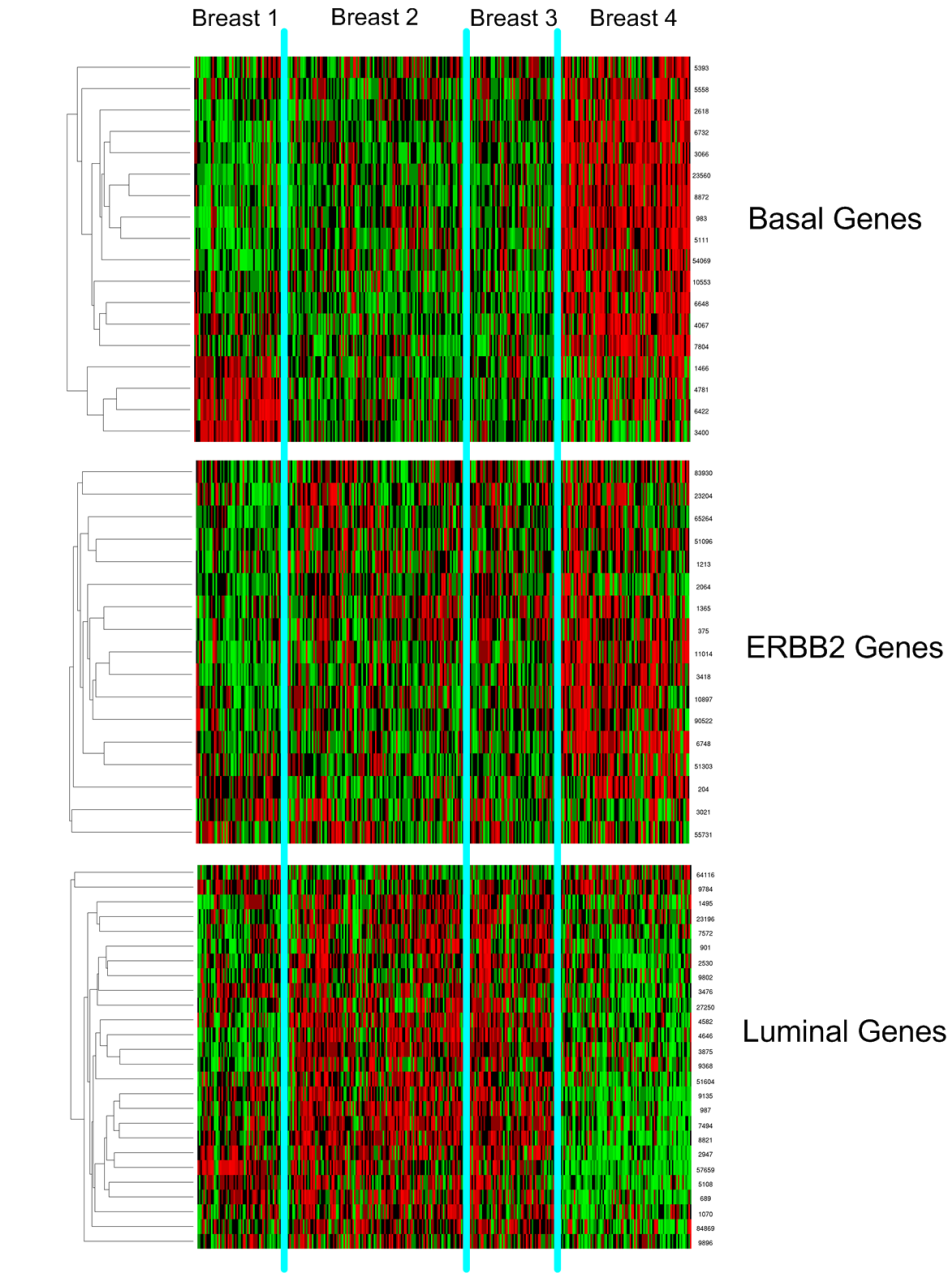
**Clustering with intrinsic genes**.

We also compared our clustering with the intrinsic breast cancer subgroups initially described by Perou *et al*. [6] and later validated in many independent data sets [4,9,44]. This analysis showed that Cluster 1, 2 and 3 largely correspond with the luminal subgroup while cluster 4 expresses both the basal and the ERBB2 (also called HER-2/neu) genes (See Figure 8). This corresponds with previous research implicating the classical, non-pleomorphic lobular and low grade ductal tumors in the luminal subgroup [37] whereas the basal tumors are associated with high grade ductal tumors [39]. The reason why we did not find a separate ERBB2-cluster might be related to the fact that the intrinsic gene list is based solely on breast tumors, while our clustering is based on genes that show variance between all types of epithelial tumors, or in other words our gene list is both intrinsic and extrinsic. In addition, also in the original clustering both the ERBB2 and basal cluster are related to each other [6,44].

In addition, a few of the most differentially expressed genes such as CAV1 and CAV2 have recently been shown to have prognostic predictive power [45,46]. More specifically, high expression of CAV1 and CAV2 in stromal cells of breast tumors is associated with a more favorable prognosis [47], which is in line with their high expression in our good prognosis cluster. Moreover, there is evidence that CAV1 expression is inversely correlated with progression of ductal carcinoma in situ (DCIS) to invasive breast cancer [48] and several recent studies highlighted the important role of the stroma surrounding DCIS in the progression to invasion [49,50]. Unfortunately, validation of our signature to the pre-invasive stage was not possible, since there are no public available gene expression data sets consisting of non-microdissected DCIS samples with follow-up data.

Also, reduced expression of proteoglycans has been associated with poor outcome and also in our signature the leucin rich small proteoglycans decorin (DCN) and fibromodulin (FMOD) are overexpressed in the good prognosis cluster [51]. This further underscores the prognostic significance of stromal gene expression in breast tumors, which is a concept that has only recently emerged [52-54].

### Lung cluster

The lung cluster is highly enriched for squamous cell carcinoma of different tissues, while such a phenomenon is not observed for adenocarcinoma. As shown in the result section adenocarcinomas cluster mainly according to their tissue of origin. It is indeed well known among pathologists that there are currently no immunohistochemical markers for the determination of the likely site of origin of squamous carcinoma, while this is in most cases possible for adenocarcinomas [55]. Our results indicate that this problem is not related to the lack of appropriate antibodies for immunohistochemical staining, but due to the absence of a molecular signature in these tumors reflecting their tissue of origin. Further attempts to identify such antibodies therefore seem useless.

### Thyroid/Kidney cluster

It has been known for quite some time that cortical tubuli in end-stage kidney diseases frequently show a morphology resembling thyroid follicles [56]. More recently, some cases of thyroid follicular carcinoma-like tumors of the kidney have been reported. This type of tumor is morphologically indistinguishable from follicular thyroid carcinoma and does not represent a kidney metastasis of a thyroid tumor. [57,58]. The strong molecular connections between thyroid tumors with follicular differentiation and chromophobe renal cell carcinomas in our study indicate that thyroid follicular carcinoma-like tumors indeed exist and probably represent a special variant of chromophobe renal cell carcinoma. Although confirmation is needed, this implies that this rare type of tumor should be clinically considered and treated as a chromophobe renal cell carcinoma.

### Ovary-endometrium cluster

The ovarian cluster segregates into two subclusters, one enriched for the endometrioids also containing the primary endometrium tumors and a cluster enriched with the serous tumors also enriched with the ovarian metastases. This clustering confirms the well-known link between ovarian endometrioid tumors and endometrioids originating from the endometrium [59] because these tumors are thought to arise from benign endometrium epithelial tissue either through endometriosis or metaplasia [60]. Serous tumors on the other hand are thought to arise form surface epithelium and usually present in more advanced stage, which explains the rather high proportion of metastasizing serous tumors in this subcluster. Our findings in this cluster clearly show that our approach is able to recover previous research findings, which indirectly increases the validity of our new findings in this study.

### Colon cluster

Besides the enrichment of primary colon tumors, this cluster was also enriched with other gastrointestinal tumors such as rectum, rectosigmoid, stomach and small intestine tumors. In addition, two subclusters emerged from the data related to clinico-pathological characteristics. The Colon A cluster clearly looks much more aggressive than Colon B. In addition no differences were found for tumor stage and histology, although mucinous histology in colon cancer has been reported as a prognostically unfavorable feature in several studies. However, a more recent analysis of a large population-based data set indicated that there is no difference in stage-specific survival between mucinous adenocarcinoma and classical adenocarcinoma [61]. The fact that mucinous carcinoma did not show a preference for either of the two clusters supports the findings of this study.

### Metastases

The bimodal nature of the tissue specificity of some metastatic tumors may offer an explanation why it is not possible for a specific subgroup of tumors to predict the tissue of origin. Breast, lung, cervix, endometrium, stomach and ovarian metastases cluster significantly in their respective primary tissue clusters while gastrointestinal metastases such as colon, rectum and rectosigmoid cluster with their tissue of destination.

Ovarian metastases occur mostly in the peritoneal cavity, most likely after losing cell adhesion processes [59,62]. This process is rather different compared to processes underlying distant metastasis via blood and lymphatic vessels and can (most likely) account for the conservation of ovarian specific expression signatures in these metastases. Breast metastases on the other hand do metastasize to distant organs, but cluster together with their primary tissue. This has also been shown in other studies, more specifically the 70 gene prognosis profile for predicting breast cancer prognosis has been shown to be conserved in breast cancer metastases [3].

Metastases of gastrointestinal origin surprisingly showed a bimodal distribution. Approximately 50% clusters together with its tissue of origin while the remaining tumors cluster in the tissue of the metastatic site. Due to the size of the colon-to-liver subset, we focused on these tumors to investigate this phenomenon in more detail and showed that these findings were supported by GSEA. Moreover, when focusing on the genes differentially expressed between the colon and liver looking metastases the A2M gene is one of the most differentially expressed genes. A2M is an acute phase reactant produced by hepatocytes, but it has been shown in a rat model that this gene is also strongly expressed in liver metastases of colon cancer [63]. Furthermore, this gene has been shown to be a marker of pre-neoplastic and neoplastic primary liver lesions [64]. In addition, others have shown that colon-to-liver metastases express liver specific RNAs and that this is due to the interaction of metastatic cells with the liver microenvironment [65]. These findings together with our data indicate that a proportion of colon adenocarcinomas that metastasize to the liver adopt hepatic features, which suggest that they represent an aggressive form of metastasis since they respond to signals from the hepatic micro-environment.

We validated this bimodal behaviour in three external data sets containing colon-to-liver metastases [66-68] by clustering the samples in each external data sets with the genes differentially expressed between both colon-to-liver subgroups. In all three data sets, the first split of the hierarchical tree was significantly enriched according to the up/down regulation in the original signature (Fisher exact test p-values < 2.2e-16).

For metastases originating from other gastrointestinal tissues the number of samples is too small to make any conclusions. However, our results support large and more detailed studies of these primary and metastatic tumors to investigate if this bimodal behavior can be generalized to all gastrointestinal tissues.

## Conclusion

The expO data set provides a unique opportunity to compare the expression profiles of many different tissues of both primary and metastatic tumor samples. In addition, extensive clinicopathological data is available, making it possible to link subgroups of tumors with clinicopathological characteristics such as histology, stage and grade. Many previous attempts in meta-analysis were limited due to different technological platforms, experimental set-up (e.g. one channel vs. two channel) or normalization methods [69]. In addition, there is still a lack of accurate and complete reporting of microarray data of cancer tissue samples. In many cases preprocessed data are reported instead of raw data making it in many cases prohibitive to use these data for meta-analysis. Moreover, phenotypic characterization of tumor samples is in many cases incomplete or even lacking while phenotypic information is crucial in the reporting of any omics data [70,71]. In the expO data set these problems are not present such that we can assume that our results are not confounded with the above mentioned issues.

An important caveat of our analysis is that due to the clinical setup of the expO study significant biases in sample selection are present. For example colorectal metastases resection is often performed in patients demonstrating metastases confined to the liver while patients with diffuse metastases are in most cases treated palliatively and are most likely not represented in the expO study. These issues however are not unique to the expO study and are also present in many of the abovementioned studies.

Our results show that distinct clusters exist corresponding to the main tissues of epithelial human cancers. In addition, similar tissues cluster together, such as tumors arising from gastrointestinal and gynecological origin. Next, breast tumors subclustered according to their main histological groups and grade. Moreover, we were able to validate a prognostic signature relevant for disease specific survival based on an unsupervised analysis in 539 patients. This prognostic signature had significant overlap with the GGI but we also found that genes related to stromal expression signatures were an important part of this prognostic signature.

Next, we also found compelling evidence that chromofobe renal cell carcinomas have overlapping gene expression features with follicularly differentiated thyroid carcinomas. Therefore, the recent morphologically defined entity of thyroid follicular carcinoma-like kidney tumors should probably be considered and treated as chromophobe carcinoma.

In addition, we also found that, in contrast with adenocarcinoma, the majority of squamous cell carcinoma cluster together irrespective of their primary tissue, supporting the immunohistochemical observation that squamous cell carcinoma do not reflect their primary tissue expression profile.

Finally, we investigated the relationship of metastatic tumors with their tissue of origin and metastatic site. Most metastases cluster with their tissue of origin. This was the case for metastases arising from breast, lung, cervix, endometrium, stomach and ovary. In the case of ovarian metastases this can be expected since ovarian metastases are thought to arise by loss of cell-cell adhesion whereas the main tissue expression profile remains the same. Lung and breast metastases on the other hand prefer more distant sites for metastasis but still cluster with their tissue of origin.

Another group of metastases, originating from gastrointestinal tissue showed a bimodal distribution, either resembling tissue of origin or tissue of destination. More specifically, colon-to-liver metastases, the largest group, showed this interesting pattern, also confirmed with GSEA analysis.

Whether colon-to-liver metastasis that respond to the liver micro-environment by expressing liver-specific genes are also more responsive to adjuvant chemotherapy is an important question. This issue appears worthwhile to be evaluated in a translational arm of a clinical study by assessing the expression of liver-specific genes by PCR of immunohistochemistry, followed by correlation with tumor regression on imaging performed during adjuvant treatment given before resection.

We believe that our taxonomy of epithelial cancers has implications on many fronts. We have shown relationships with clinical outcome, discovered new subgroups, identified a squamous expression profile over multiple tissues and studied the relationship between primary and metastatic tumors. These findings will provide important information for pathologists interpreting histological slides, researchers investigating CUP and the development of prognostic signatures for breast cancer.

## Competing interests

The authors declare that they have no competing interests.

## Authors' contributions

OG, AD, BDM and LL conceived the study, participated in the analysis of the data and writing of the manuscript.

## Pre-publication history

The pre-publication history for this paper can be accessed here:

http://www.biomedcentral.com/1755-8794/2/69/prepub

## Supplementary Material

Additional file 1 **Sample list**. List of all samples in each of the molecular clusters. Click here for file

Additional file 2 **GSEA analysis all clusters**. Upregulated gene sets in each cluster vs. the remaining samples according to GSEA analysis. Click here for file

Additional file 3 **Breast cancer clinico-pathological data**. Grade, histological distribution and receptor status for the breast subclusters. Click here for file

Additional file 4 **Breast signature**. Breast signature based on differentially expressed genes between Cluster 1 and Cluster 4 in the breast cluster. Click here for file

Additional file 5 **GSEA kidney samples**. Upregulated gene sets in the primary kidney samples in the Thyroid-Kidney cluster vs. the primary kidney samples in the Kidney cluster according to GSEA analysis. Click here for file

Additional file 6 **GSEA colon-to-liver metastases**. Pathways from the colon-to-liver metastases upregulated either in the colon or in the liver cluster according to GSEA analysis. Click here for file

Additional file 7 **Tissue specific genes**. Comparison of the kidney and liver expression signatures from the TIGER database. Click here for file

Additional file 8 **Differentially expressed genes in breast cluster**. Differentially expressed genes between the lobular primary breast samples and the ductal primary breast samples in the mixed lobular-ductal cluster (Cluster A). Click here for file

Additional file 9 **DAVID results**. DAVID functional enrichment analysis of the Breast signature separately for the genes expressed in Cluster 1, Cluster 4 and the GGI signature. Click here for file
